# Visceral Nociceptive Afferent Facilitates Reaction of Subnucleus Reticularis Dorsalis to Acupoint Stimulation in Rats

**DOI:** 10.1155/2013/931283

**Published:** 2013-05-15

**Authors:** Liang Li, Lingling Yu, Peijing Rong, Hui Ben, Xia Li, Bing Zhu, Rixin Chen

**Affiliations:** ^1^Institute of Acupuncture and Moxibustion, China Academy of Chinese Medical Sciences, Beijing 100700, China; ^2^Wuhan Integrated TCM and Western Medicine Hospital, Wuhan 430020, China; ^3^Jiangxi University of Traditional Chinese Medicine, Nanchang 330004, China

## Abstract

*Objective*. To explore the area and sensitization variance of acupoint when internal organs are under pathological condition. To observe quantity-effect variance of subnucleus reticularis dorsalis (SRD) to electroacupuncture under both physiological and pathological conditions. To explain medulla oblongata mechanism of acupoint sensitization. *Method*. Mustard oil was imported into colon and rectum of 20 male SD rats in order to observe its influence on acupoint sensitization. SRD neuron activity was recorded. Visceral nociceptive stimulus was generated by colorectal distension (CRD). Quantity-effect variance of neuron activity to electroacupuncture to “Zusanli-Shangjuxu” area both before and after CRD was observed. Paired *t*-test is used for cross-group comparison; *P* < 0.05 is deemed as of statistical differences. *Result*. Visceral inflammation could facilitate SRD neuron activity to acupoint stimulation. Visceral nociceptive afference could enhance neuron activity to acupoint acupuncture. Wide dynamic range (WDR) neuron activity caused by electroacupuncture increased when visceral nociception increased. *Conclusion*. The size and function of the acupoints comply with the functionality of the internal organs. The sensitive degree of acupoints changed according to malfunction of internal organs.

## 1. Introduction

The theory ofacupoints has been matured through the development of history. Yet the essence of the acupoint theory remains “selection of the pain point as an acupuncture point” (points with the sense of relief or pain upon pressing), that is, the *ashi* points. Those pain points, in fact, are typical peripheral points that reflect the pathological changes to the correlated organs. It covers the two functions of acupoint as diagnosis (pain upon pressing) and treatment (sense of relief upon pressing) of diseases. Acupoints are windows that reflect specific changes in the internal organs. Points for analgesia contain many features resembling referred pain in western medicine. Referred pain caused by inner organs usually accompanies hyperalgesia of the skin [1] or muscle and often the occurrence of segmental muscle contracture [2, 3]. This kind of acupoint reaction is similar to hyperalgesia and allodynia which appeared due to sickness of inner organs. Hyperalgesia refers to the overreaction of algesia that occurres in our bodies in case of stimulation, which includes pain triggered by subthreshold stimulus that should not cause pain when the body is in its normal condition and severer pain triggered by suprathreshold stimulus that in a normal case would not cause as much pain. Allodynia refers to pain caused by situations which in the normal case should not cause pain. Allodynia is actually a kind of hyperalgesia [4]. For instance, it has been observed that a slight touch on the body surface or hair can trigger pain in the referred pain area [5]. The reason for this kind of “pain upon pressing” actually involves mechanism of hyperalgesia or allodynia in the referred area correlated to certain inner organs.

Pathological changes in the inner organs can be manifested on the surface of the body and cause the sensitization of acupoints with various kinds of pathological reactions mainly led by pain. The reason that pathological changes can achieve the above is that they are the specific reflections of the inner organs manifested through the acupoints. To some degrees, acupoints are capable of reflecting the pathological conditions of organs and viscera. By examining changes in the feeling, shape and color of acupoints, diagnosis of inner organs can be made. In the meantime, acupuncturing on the points serves the purpose of treating the inner organs by dredging the meridians and regulating *qi* and blood. Therefore, superficial reflection areas where inner organ sicknesses manifest should be the most desirable spots to perform acupuncture so as to regulate the functionality of inner organs thus treating inner organ diseases. 

Currently, the neurobiological mechanism of the sensitization of acupoints remains unclear. However, when inner organs are sick, referred pain converges. According to the facilitation theory, the diseased organ and part of the skin where referred pain occurs are both innervated by the afferent fibers from the dorsal spinal nerve, both of which end in the same area in the dorsal horn. As a result, when inner organs are sick, more impulse is sent to the dorsal horn, creating an excitation spot, thus leading to the decreasing of the threshold. Studies on referred pain have preliminarily revealed that body-viscera convergent neurons in the spine and the body of central supraspinal can be sensitized by the stimulation that comes from the inner organs, rendering that convergent neurons react stronger to the input from the body surface [6–8]. This also serves as reliable scientific proof for acupoint sensitization.

Electrophysiologic research reveals that subnucleus reticularis dorsalis (SRD) that locates in the caudal portion of the medulla can be activated by nociceptive mechanical, thermal, and chemical stimulation that comes from different parts of the body [9]. One of the most important features of the SRD neuron is that harmful information that comes from various parts of the body will gather in one neuron. Secondly, what is important is that SRD neurons have spatial summation ability against harmful stimulation: to a certain extent, activation reaction increases with the expansion of stimulating area; once it reaches or exceeds a certain area, the reaction of neurons reach saturation [10], some may even decrease. These data illustrate the occurrence of convergence between the afferent signals of the body and organs.

Discovering the rules and mechanism of the acupoint sensitization with diseased inner organs can provide guidance for clinical acupoint selection and enhance the efficacy of acupuncture in treating inner organs. When organs are sick, superficial acupoints are in different states of “sensitization” and “silence,” thus altering the “quality” and “quantity” in the toning or treatment of organs. This research will study on the dynamic variation rules of acupoints from relatively “silence” to “active” state at SRD level. The correlation and other relating mechanism between the sensitization of acupoints and functionality of inner organs are also discussed. 

## 2. Materials and Methods

### 2.1. Experiment Animals and Method

Experiments were performed on 20 Sprague Dawley rats (between 250 and 300 g). Following an intraperitoneal injection of 100 *μ*g atropine sulfate, the animals were anesthetized with an intraperitoneal injection of urethane (1.0~1.2 g · kg^−1^). A tracheal cannula was inserted and the animals were paralyzed by intravenous injection of gallamine triethiodide (Flaxedil) and artificially ventilated. Heart rate was continuously monitored and core temperature as maintained at 37 ± 0.5°C by means of a feedback-controlled homeothermic heating blanket system.

The animals were mounted in a stereotaxic frame with the head fixed in a ventroflexed position by means of a metallic bar cemented to the skull, and the caudal medulla was then exposed by removing the overlying musculature, atlantooccipital membrane, and dura mater. 

Unitary extracellular recordings were made with glass micropipettes filled with a mixture of 2% pontamine sky blue and 0.1 M of natrium aceticum (cusp: 5 *μ*m, impedance: 8–12 MΩ).

Single-unit activities were recorded extracellularly and the isolated action potentials were fed into a window discriminator and displayed on an oscilloscope screen. The output of the window discriminator and amplifier were led into a data collection system (PowerLab) and a personal computer data acquisition system (Chart 5.2) to compile histograms or wavemark files.

The micropipettes were inserted on the left side of the medulla, 1.0–2.0 mm caudal to the obex, and 0.5–1.5 mm lateral to the midline. Stability for the recordings was achieved by placing over the surface of the medulla 2% Ringer-agar gel. Nonnoxious and noxious electrical or mechanical search stimuli were used to help isolate unitary activity, and neurons were classified on the basis of their responses to different stimuli applied to their peripheral receptive fields. The neurons that could be activated by noxious stimulation applied to every part of the body, and internal organs were identified as SRD neurons.

For visceral-intrusive inflammatory reaction, catheterization injection was adopted. A conduit was inserted into the rat's colorectum through anus with a depth of 2-3 cm. Then 20 *μ*L of 2.5% mustard oil was injected (Sigma-Aldrich, St. Louis, MO, USA) through the conduit. 

Visceral-intrusive stimulation is done by colorectal distension. A condom was used to make a 4–6 cm-long air sac, and it was tied to a 4 mm-diametered rubber tube. The tube was connected to a sphygmomanometer-pressure transducer with a T-tube. During the experiment, the air sac was inserted into the rat's colorectum with a depth of 4 cm. CRD stimulation is carried out by pressure supplied by a 20–80 mm Hg sphygmomanometer for 20 s or longer. Previous research [11, 12] indicated that the pressure bigger than 40 mm Hg is visceral-intrusive stimulation. In order to prevent possible sensitization triggered by over stimulation in the colorectum, the interval between two CRD stimulations should be at least more than 10 minutes.

### 2.2. Experiment Procedure

(1) First, response of SRD neurons to electroacupuncture at Zusanli (ST36) and Shangjuxu (ST37) in 20 rats before and after the introduction of mustard oil (20 *μ*L) was compared in order to observe if visceral inflammatory reaction can facilitate the response caused by electroacupuncture.

(2) The stimulus intensity of electroacupuncture at Zusanli (ST36) and Shangjuxu (ST37) is 1.5 times stronger than the threshold of A*δ* fiber reflection (please refer to our previous work for detailed operation [13]. The average intensity of A*δ* fiber reflection threshold is 1.77 ± 0.53 mA). The pressure of CRD was 20, 40, 60, and 80 Hg. The dose-effect relationship between visceral-intrusive stimulation and SRD neuron sensitization in various levels; nonintrusive (20 mm Hg), mild (40 mm Hg), moderate (60 mm Hg), and strong (80 mm Hg), was observed.

### 2.3. Histological Position of the Recorded Sites

After single-unit recording, the location of the recording site was checked by HE coloration. By referring to the brain atlas of the rat (Paxinos and Watson, 2007), the data will be abandoned if the recording site is out of the SRD neurons area.

### 2.4. Data Collection and Analysis

Neurons discharges per second and the activation/suppression ratio (identify as $\overset{-}{X}\pm \text{SE}\%$) were calculated with Power-Lab, Chart 5.0, and SPSS13.0. Descriptives were carried out for the average and differences of the pre- and postintervention (identify as $\overset{-}{X}\pm \text{SE}$). Paired *t*-test is used for cross group comparison. *P* < 0.05 is deemed as of statistical differences.

## 3. Results 

### 3.1. General Features of SRD Neurons

**Figure 1:**
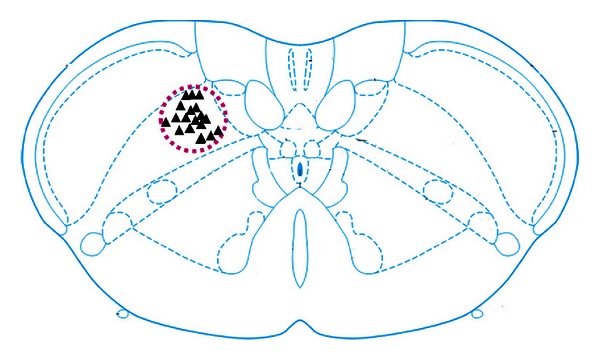
▲ indicates the pontamine sky blue positioning of SRD neurons.

Totally, 65 neurons were recorded in the dorsal medulla in 20 SD male rats, among which 58 were SRD neurons and 7 were neurons from the spinal nucleus of trigeminal nerve.  Figure 1 illustrates part of the pontamine sky blue positioning of SRD neurons.

Any suprathreshold electric stimulation at any part of the rat's body is capable of activating SRD neurons. Stimulation at the tale tip or 10 cm from the tip in the basilar area could trigger two peaks in the activation reaction. The latency period of the two peak reactions in the basilar area remains 14~45 ms and 185~260 ms respectively. The latency period of the two reactions in the tale tip area is 22~58 ms and 545~670 ms, respectively. The time difference in the early stage of activation reaction peak between the two areas is 10.5 ± 0.7 ms. Calculations suggest that the conduction velocity of the peripheral fibers is 12.5 ± 1.3 m/s, according with the conduction velocity range of A*δ* fibers. The time difference in later period in activation reaction peak between the two basilar areas is 152 ± 14.4 ms. Calculations suggest that the conduction velocity of the peripheral fibers is 0.78 ± 0.13 m/s, in accordance with the conduction velocity range of C fibers. Therefore, an inference can be made that suprathreshold electric stimulation can activate A*δ* and C fibers [10]. 

No SRD neuron reacted to any kind of nonintrusive stimulations (such as sound, light, and proprioceptive stimulus). SRD neurons had significant reactions towards general intrusive mechanical stimulations (e.g., pinching on the skin with toothed forceps) or 48°C water stimulation, and so forth. 

### 3.2. Activation Effect of CRD on SRD Neurons

**Figure 2:**
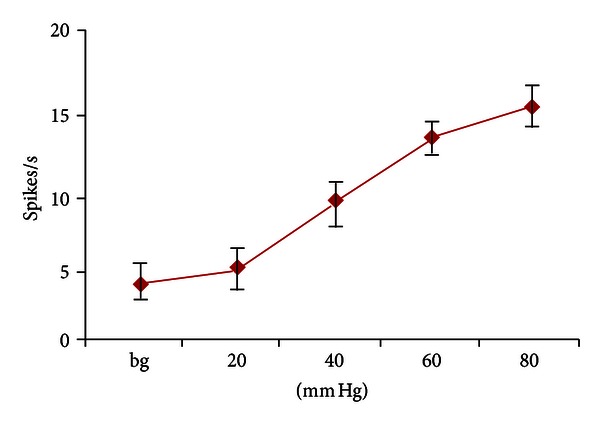
The activation effect of 20–80 mm Hg CRD on SRD neurons.

In the experiment, we examined 9 SRD neurons on their reactions to 20–80 mm Hg CRD stimulations. The degree of activation increased to 4.42 ± 0.68 spikes/s (*P* < 0.05) from 3.55 ± 0.63 spikes/s with 20 mm Hg of CRD; when CRD was set at an intrusive level of 40 mm Hg, the degree of activation of SRD neurons reached 8.80 ± 1.13 spikes/s (*P* < 0.001); when imposed with 60 and 80 mm Hg of CRD, the degree of activation of SRD neurons reached 13.27 ± 2.82 and 15.11 ± 2.63 spikes/s, both of great statistical significance (*P* < 0.001). This indicates that visceral-intrusive damage could activate the activity of SRD neurons in a scale-differentiating manner, and is of significant dose-effect relation (Figure 2).

### 3.3. The Effect of Acupoint Sensitization by Inserting Mustard Oil into Colorectum on SRD Neurons

Activity of 18 convergent neurons was recorded in 20 rats which could be activated by CRD and stimulation at Zusanli-Shangjuxu area. In normal cases, spontaneous activity was rarely seen among these neurons. After insertion of 20 *μ*L mustard oil through the colorectal conduit, spontaneous activities of the SRD neurons was significantly increased (from 3.42 ± 0.64 spikes/s before insertion and 6.04 ± 1.63 spikes/s after; *P* < 0.01). 

**Figure 3:**
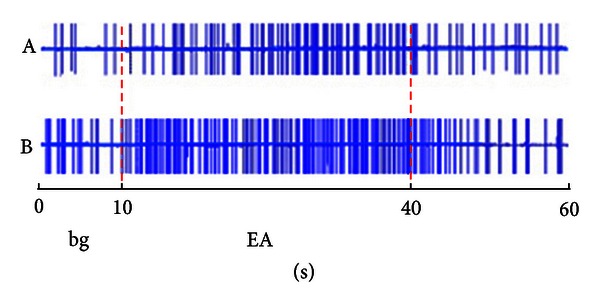
Differences in activation of SRD neurons before (A) and after (B) mustard oil insertion in the colorectum (indicating internal organ inflammatory reaction facilitates afferent from acupoints).

By using stimulation of 1.5 times stronger than A*δ* fiber reflection threshold at Zusanli (ST36) and Shangjuxu (ST37), activity of 18 SRD neurons were observed before and after the colorectal insertion of the mustard oil. Before the insertion, discharge activity increased from 4.13 ± 0.77 spikes/s to 6.05 ± 1.42 spikes/s. While after injection of mustard oil, electroacupuncture at Zusanli could increase neuron discharge by 46.48 ± 11.45%. This indicates that electroacupuncture could activate SRD neurons with statistical difference (*P* < 0.05). After the insertion of mustard oil, on the other hand, SRD neurons were significantly activated by EA (*P* < 0.01). The activity of SRD neurons increased to 10.47 ± 2.23 spikes/s, which is 70.86 ± 15.48% increasing compared with non-EA. The activation effect of EA stimulation with the same intensity on SRD neurons showed an increase of 73.05 ± 14.22% compared to before the insertion of mustard oil (*P* < 0.01) (Figure 3).

### 3.4. Effect of Acupuncture on SRD Neurons and Its Relation with CRD

This part of the research is to explore whether continuous nociceptive visceral afferent can trigger sensitization for relating acupoints and the medulla mechanism of acupoint sensitization. 20–80 mm Hg of CRD was carried out on the rat for 30 s to trigger activation of SRD neurons. Then after CRD, changes of SRD neurons activity to electroacupuncture at Zusanli-Shangjuxu area which is 1.5 times stronger than A*δ* fiber reflection threshold are observed before and after CRD stimulation respectively. 

**Figure 4:**
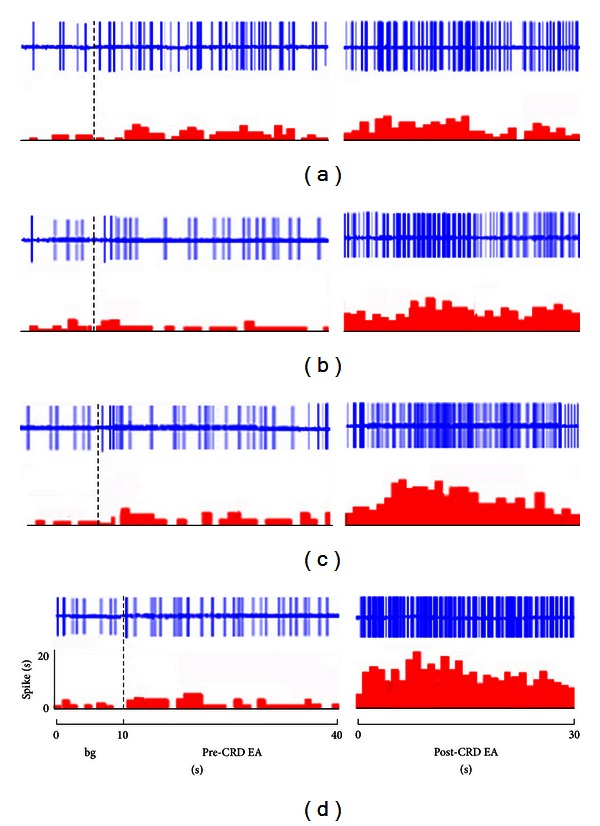
The responses of SRD neurons to EA before and after CRD ((a) 20, (b) 40, (c) 60, and (d) 80 mm Hg, resp.). Note: upper rows showing original unit discharges and lower rows showing histograms.

The activity of SRD neurons increased with CRD pressure increasing in the range of 20–80 mm Hg for 30 s. The activity of SRD neurons increased significantly to EA stimulation after CRD (see Figures 1 and 4). This indicated that acupoint sensitization occurred after continuous nociceptive CRD stimulation was performed on the rat (see Table 1). 

Activity of 16 SRD neurons to EA stimulation 1.5 times stronger than A*δ* fiber reflection threshold was observed both before and after 20 mm Hg CRD.  Figure 4 indicates that before CRD of 20 mm Hg, EA stimulation could significantly activate SRD neurons. Compared to the background, the activation percentage of neurons caused by EA was 80.92 ± 7.84% (*P* < 0.001). After CRD, EA activation to SRD neurons increased 113.10 ± 10.92% compared to its effect before CRD (*P* < 0.05).

**Figure 5:**
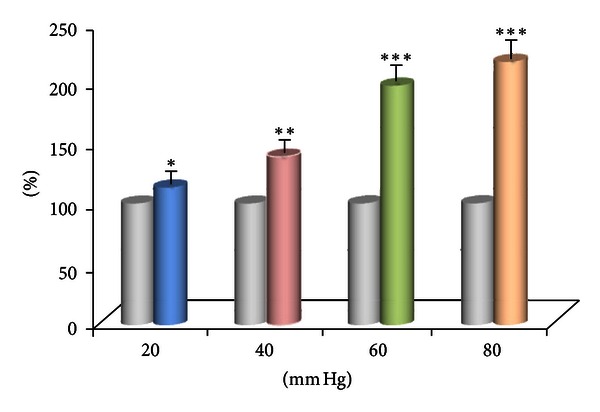
The response of SRD neurons to EA at different CRD levels (Left: before; Right: after). The response of SRD neurons to EA increases with CRD and shows a distinct dose-effect relation (*P* < 0.05~0.001).

Activity of 16 SRD neurons to EA stimulation 1.5 times stronger than A*δ* fiber reflection threshold was observed after 40 mm Hg CRD. The intensity of activation caused by EA increased by 38.35 ± 5.12% after CRD, illustrating significant statistical differences (*P* < 0.001). After the intensity of 60 and 80 mm Hg of CRD, the degree of activation of SRD neurons caused by EA increased by 96.49 ± 11.02% and 116.10 ± 12.89% both of which indicating significant statistical significance (*P* < 0.001). From 20 mm Hg to 80 mm Hg, the facilitation effect of CRD to EA activation effect on SRD neurons increases with pressure increasing. There is a linear relation between the two effects (Figure 5).

These results indicate that nociceptive internal organ distention is capable of sensitizing SRD neurons in the medulla which renders its reaction to the EA at acupoints becoming stronger. There is a linear relation between intensity of nociceptive stimulation to internal organ and sensitization of related acupoint. All of the results above demonstrate that SRD participates in the dynamic change of the sensitization of acupoints.

## 4. Discussion

As we have observed in the experiment, EA is capable of activating the activity of SRD neurons. Yet after CRD was performed, SRD neurons showed stronger reaction to the same intensity of EA at the same acupoint. This reveals that CRD is capable of sensitizing SRD neurons.

Previous studies on the relation of acupoint and organs mostly focus on the acupoints' regulation of the function in healthy state neglecting the fact that the function and area of the acupoints could vary under pathological circumstance. In recent years, we put forward the concept of “dynamics states of acupoints,” deeming that the size and function of the points are not in a stable state but a changing and dynamic one. The function and size of acupoints will vary along with the state of the body, especially with the function of internal organ [14]. This experiment reveals the relation of reaction points and acupoints in organ-diseased rats. A morphological study shows that under inflammatory state, sick organs can promote the secretion of Evans blue on the body surface, and those seepage points are related to acupoints on the relating meridians. For rats with ovarian inflammation, their seepage points mainly distribute around “Guanyuan (RN4)”—“Uterus” area, and “Shenshu (BL23)”—“Mingmen (DU4)” follows [15]. For rats with acute gastric mucosa inflammation, the seepage points aligns with nerve segments and are highly related to “Pishu (BL20),” “Shenshu (BL23),” and so forth [16]. During our medulla experiment, we discovered that when WDR is activated by intrusive CRD, giving EA in the receptor filed will trigger further activation of WDR neurons. This indicates that peripheral and afferent nerves that derive from the same nerve segments meet at the WDR, thus illustrating concerted reaction. Another part of our experiments shows that after ending long time intrusive CRD stimulation, giving the same intensity of EA in the receptor field causes a stronger activating reaction in WDR and SRD neurons as compared with before the CRD, indicating that CRD has managed to increase the sensitization of neurons. 

These studies have shown that along with the changes from healthy to pathological state of the organs, the function of acupoints could alter from silent state into an active or sensitized state becoming rather sensitiveand active.

It is a central mechanismfor acupoints to reflect organ diseases that the primary afferent of the body and organs meet in the nervous centralis. Studies such as Cervero and Connell [17] and Cervero [18] showed that the intercostal nerve and greater splanchnic nerve of the cat convergein the thoracic cord. With light and electron microscope, Jishuo and Binzhi [19] have discovered that visceral primary afferent of the pelvic nerve and corporality primary afferent of the sciatic nerve both project to the sacral dorsal commissural nucleus. He has also found that some of the two afferents converge into the same dendrite of the dorsal commissural nucleus. Alles and Dom [20] injected dual-marking fluorescence indicator into the inner side of the arm and pericardium and found the indicator in the same side of dorsal root ganglion at C_8_-T_2_. The sensory nerve projection of the rabbit's riyue and qimen area shares 5–7 overlapping segments with the sensory nerve in the choledoch ampullary portion [21]. These studies not only prove the convergence of the peripheral and organ afferent but also serve as direct morphological proof inreferred pain in western medicine.

The abnormal hyperalgesia occurance upon pressing acupoints while the internal organs are sick is the result of facilitation and sensitization of the spinal cord and/or supraspinal centrum while pathological changes take place in the organs and is in accordance with the converging sensitization/facilitation mechanism that explains referred pains. Studies have shown [6, 22] that afferent impulse in the organs or deep tissues could sensitize the body-organ convergent neurons in the dorsal horn of the spinal cord, thus rendering that neurons have intensified reaction to the afferents that come from the body surface. After these convergent neurons have experienced sensitization caused by visceral changes (intrusive stimulation), the number of spontaneous discharging cells increases, the frequency of discharging goes up, the threshold of stimulation decreases, and the surface receptor field expands. For instance, for animals with referred muscle hyperalgesia caused by ureteral calculus, the number and frequency of background discharging spinal cord cells exceed that of the normal animals [7]; after chemical stimulation in the bladder, the background discharging of neurons in the dorsal horn of the spinal cord elevates [6]; compared with normal rats, rats with ureteral calculus have a greater number and frequency of background discharging in neurons from the dorsal horn of the spinal cord; the author considers this as referred hyperalgesia [8]. Besides, inflammation in the esophagus [23] and colon [24] may lead to the reduction in reaction threshold. Multiple distending with air sac in the esophagus results in the expansion of the receptor field in dorsal horn neurons in T_2–4_. By the same token, distending gall bladder with 65–80 mm Hg can lead to the expansion in receptor field for the skin in body-organ convergent neurons in the dorsal horn of the spinal cord. Injecting glutamate in SRD could facilitate the activation reaction of WDR on EA sciatic neurons [25]. 

## 5. Conclusions

The convergent sensitization mechanism refers to the reduction of reaction threshold, increasing of background discharging, and the expansion of receptor filed when body-organ convergent neurons experience pathological changes in the organs. This has shown that the size and function of the acupoints comply with the functionality of the internal organs. Those studies have provided scientific proof for acupoints status transforming from silence to activation.

## Figures and Tables

**Table 1:** The firing discharges of SRD neurons induced by EA before and after CRD stimulation (spikes/s).

CRD intensity (mmHg)	*n*	BG (spikes/s)	EA before CRD (spikes/s)	EA after CRD (spikes/s)
20	16	3.25 ± 0.27	5.88 ± 0.72	6.65 ± 0.64
40	16	3.65 ± 0.36	6.31 ± 0.68	8.73 ± 0.47
60	15	3.95 ± 0.44	5.69 ± 0.73	11.18 ± 1.42
80	17	3.77 ± 0.62	5.66 ± 0.54	12.26 ± 1.72
